# Infected necrotizing pancreatitis: clinical features, microbial patterns, and outcomes in a Saudi tertiary center

**DOI:** 10.3389/fcimb.2025.1689728

**Published:** 2025-10-15

**Authors:** Moaz Abulfaraj

**Affiliations:** Department of Surgery, Faculty of Medicine, King Abdulaziz University, Jeddah, Saudi Arabia

**Keywords:** acute pancreatitis, infected necrotizing pancreatitis, multidrug-resistant bacteria, GLP-1 agonists, antibiotic stewardship, enteral nutrition

## Abstract

Infected necrotizing pancreatitis (INP) is a severe complication of acute pancreatitis (AP) associated with high morbidity and mortality, yet regional data remain limited. We retrospectively reviewed 119 patients admitted with AP to the King Abdulaziz University Hospital, Jeddah, Saudi Arabia, between 2017 and 2025 to characterize risk factors, microbiological profiles, and management strategies. Of these patients, 21 (17.6%) developed INP. Compared with patients with noninfected AP, they were older (mean 60.1 ± 11.2 years, *p* < 0.05), were more frequently overweight or obese (85.7% ≥ 25 kg/m², *p* = 0.03), and had higher severity scores (Bedside Index for Severity in Acute Pancreatitis ≥3 in 61.9%, *p* < 0.01). These baseline differences could explain outcomes rather than infection status. Gallstones (42.9%) and exposure to glucagon-like peptide-1 (GLP-1) receptor agonists (4.8%) were associated with INP. Radiologic imaging confirmed necrosis in all cases, including five abscesses. Of the 21 INP cases, 19 were confirmed by positive cultures from fine-needle aspiration (FNA), drainage, or necrosectomy and 2 by imaging (gas in necrotic collections) plus blood cultures. Microbiological cultures obtained from FNA, drainage, or necrosectomy most commonly identified *Escherichia coli* (42.9%) and *Klebsiella pneumoniae* (23.8%), with multidrug resistance detected in one-third of cases. Management strategies included conservative therapy (*n* = 8), percutaneous drainage (*n* = 4), endoscopic necrosectomy (*n* = 5), and surgical necrosectomy (*n* = 4). Complications included pancreatic fistula, colonic perforation, and sepsis, with an overall mortality rate of 9.5%. Antibiotics were culture-directed in INP but frequently used prophylactically in severe noninfected AP. Early enteral nutrition was implemented in most infected cases. These preliminary findings—limited by a small sample size, single-center design, and a bias toward severe cases—require validation in larger studies; nevertheless, they suggest that older age, obesity, severe presentation, and multidrug-resistant Enterobacteriaceae are defining features of INP in this cohort, and they underscore the importance of antibiotic stewardship and early nutritional support in regional clinical practice.

## 1 Introduction

Acute pancreatitis (AP) affects 20–40 per 100,000 individuals worldwide, with necrotizing pancreatitis forms complicating 10%–20% of cases and carrying mortality rates of up to 30% (Banks et al., 2013; Petrov and Yadav, 2019). In Saudi Arabia, gallstones remain the leading cause of AP, but the increasing use of glucagon-like peptide-1 (GLP-1) receptor agonists for weight loss, in the context of obesity rates exceeding 35%, may represent an emerging risk factor (Faillie et al., 2016; Alkhiari et al., 2021).

Infected necrotizing pancreatitis (INP), defined by imaging or microbiological confirmation, is associated with worse outcomes than sterile necrosis and systemic inflammatory response syndrome (SIRS) (Mouli et al., 2015). Its management is further complicated by the global rise of multidrug-resistant (MDR) organisms, particularly *Escherichia coli* and *Klebsiella pneumoniae*, which are increasingly reported in the Middle East (Memish et al., 2016). Current international guidelines recommend a step-up strategy, progressing from conservative therapy to minimally invasive and surgical approaches as needed (Boxhoorn et al., 2020; Trikudanathan et al., 2019). However, regional differences in microbial profiles, antimicrobial resistance, and resource availability necessitate locally tailored protocols.

Previous regional work has highlighted gallstone-related AP as a major burden (Aljiffry et al., 2023), but comprehensive data on INP, its microbiology, and management strategies in Saudi Arabia are lacking. This study retrospectively analyzed patients with AP admitted to a tertiary care center in Jeddah to characterize the epidemiology, microbiological spectrum, therapeutic approaches, and outcomes of INP, with a particular focus on antibiotic stewardship and non-antibiotic strategies.

## 2 Materials and methods

### 2.1 Study design and population

**Figure 1:**
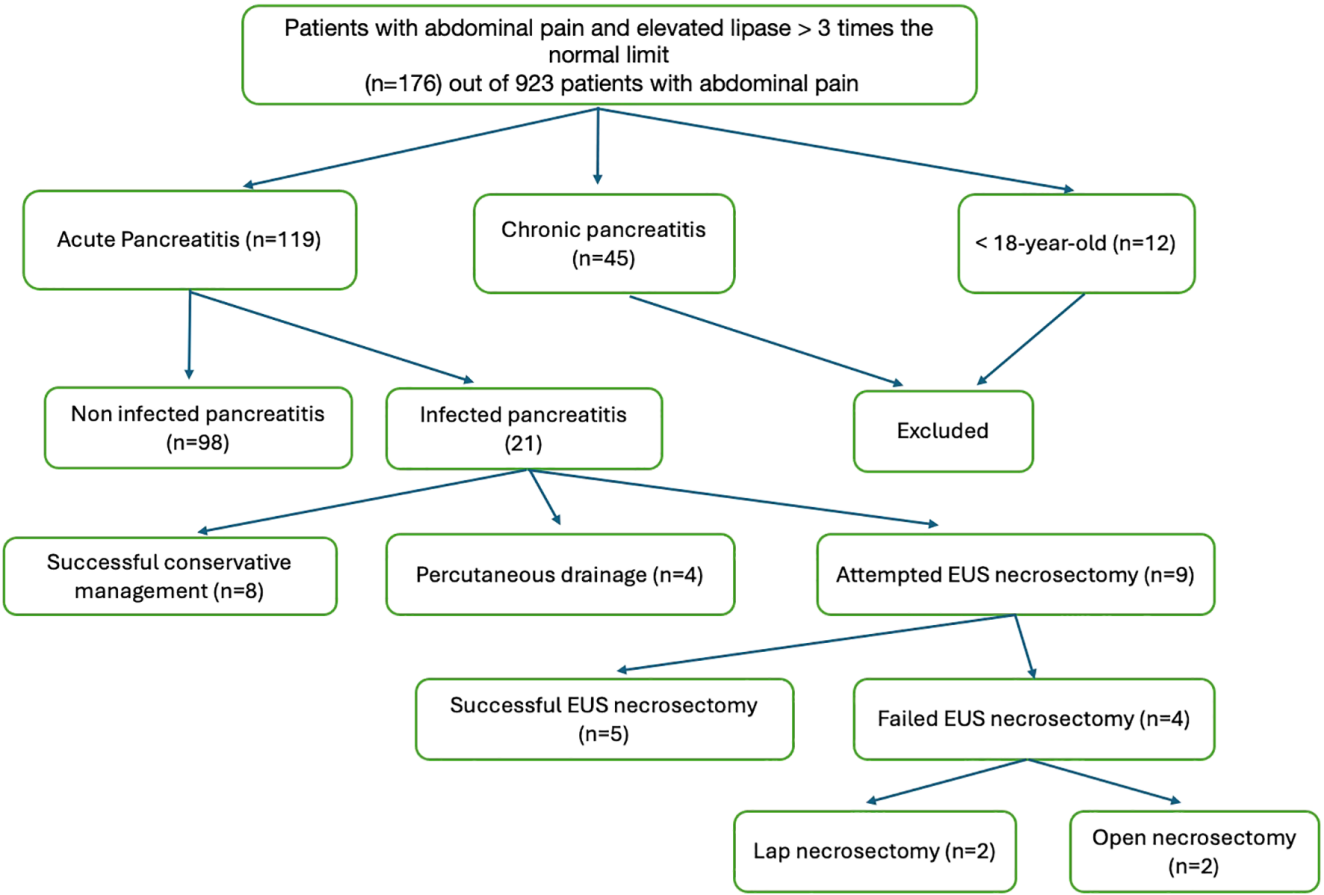
Flowchart of patient selection. Of the 923 patients with abdominal pain, 119 met the Atlanta criteria for AP. Exclusions: chronic pancreatitis (*n* = 45) and age <18 years (*n* = 12). Of the 119 AP cases, 21 had infected necrotizing pancreatitis based on imaging or cultures.

We conducted a retrospective cohort study of 119 patients with AP admitted to the King Abdulaziz University Hospital (KAUH), Jeddah, Saudi Arabia, between 2017 and 2025, and 21 of these patients were diagnosed with INP. Eligible patients were identified through emergency department records and included if they met the revised Atlanta criteria for AP, defined by at least two of the following: serum lipase or amylase ≥3 times the upper limit of normal, characteristic abdominal pain, or radiological evidence on computed tomography (CT) or magnetic resonance imaging (MRI) (Banks et al., 2013). Necrotizing pancreatitis was confirmed by imaging, and infection was established by positive cultures from percutaneous fine-needle aspiration (FNA), drainage, or necrosectomy (using sterile techniques to minimize contamination), or by radiological features highly suggestive of infection (e.g., gas in necrotic collections) in clinically deteriorating patients. Cultures containing skin flora, particularly low-virulence organisms (e.g., *Staphylococcus epidermidis*), were considered contaminated when interpreted alongside clinical assessment. Patients with chronic pancreatitis or younger than 18 years were excluded. The timing of infection diagnosis varied owing to tertiary referrals, with >33% of patients initially managed at other facilities. A flow diagram of patient selection is shown in **Figure 1**. The study protocol was approved by the KAU Research Ethics Committee.

### 2.2 Data collection

We extracted demographic data [age, sex, and body mass index (BMI)], clinical features [etiology, Bedside Index for Severity in Acute Pancreatitis (BISAP) score, admission C-reactive protein (CRP), and lipase levels], imaging findings, microbiological results, complications (infected necrosis, abscess, and colonic perforation), treatments (antibiotic use and interventions), and outcomes [length of hospital stay, intensive care unit (ICU) admission, and mortality]. Two independent reviewers collected information from electronic medical records, and discrepancies were resolved by a third reviewer. Missing data, including incomplete follow-up in 10 of 21 infected cases, were documented but not imputed due to the retrospective design. Infection was confirmed by positive cultures from FNA, drainage, or necrosectomy, or by characteristic radiological features such as intralesional gas in clinically deteriorating patients. Severity was assessed using the BISAP, with scores ≥3 defining severe disease (Wu et al., 2008). Antibiotic therapy was administered within 24 h of infection confirmation and tailored to culture results, including cephalosporins for *E. coli* and carbapenems for MDR strains. Prophylactic antibiotics (cephalosporins, carbapenems, or metronidazole/piperacillin–tazobactam) were administered in severe non-infected AP (BISAP ≥ 3 or necrosis > 30%), or when concomitant infections such as cholecystitis or cholangitis were suspected, according to local guidelines. Conservative management included fluid resuscitation, pain control, and early enteral nutrition within 48 h via nasogastric or nasojejunal tubes when feasible. Total parenteral nutrition (TPN) was reserved for patients with ileus or following interventions. Minimally invasive approaches included percutaneous drainage for abscesses and endoscopic ultrasound (EUS)-guided cystogastrostomy or necrosectomy for walled-off necrosis (>4 weeks). Surgical necrosectomy (laparoscopic or open) was performed when endoscopic treatment failed or when necrosis was solid or inaccessible. Cholecystectomy was undertaken during the index admission for gallstone-related non-infected AP or at the time of necrosectomy in infected cases.

### 2.3 Statistical analysis

Data were analyzed using the SPSS software, version 26 (IBM Corp., Armonk, NY, USA). Normally distributed data were reported as mean ± SD, and non-normally distributed data as medians. Comparisons between categorical variables were performed using the chi-squared test. Logistic regression analysis was applied to identify predictors of infection, including age, BMI, BISAP score, and hospital stay. Kaplan–Meier survival analysis was used to estimate time to infection. Ninety-five percent confidence intervals (95% CI) were calculated for complication and mortality rates. No statistical comparisons were performed for subgroups with fewer than five patients. Furthermore, because of limited subgroup sizes (*n* = 2–8), intervention categories were consolidated into four groups for some analyses: endoscopic necrosectomy, surgical necrosectomy (laparoscopic or open), percutaneous drainage, and conservative management. A two-tailed *p*-value <0.05 was considered statistically significant.

## 3 Results

### 3.1 Incidence and epidemiology

Among the 923 patients who presented with abdominal pain, 119 (12.9%) were diagnosed with AP, of whom 21 (17.6%) developed INP. Five of the INP cases (23.8%) had associated abscesses. Etiologies of INP included gallstones (9/21), GLP-1 agonist use (1/21), idiopathic causes (5/21), autoimmune disease (3/21), and alcohol (3/21). Across the full cohort (*n* = 119), the distribution was gallstones 49.6% (59/119), GLP-1 agonists 1.7% (2/119), idiopathic 22.7% (27/119), autoimmune 8.4% (10/119), and alcohol 5.9% (7/119). Two cases were linked to GLP-1 agonist exposure. One patient (female, 55 years, BMI 30 kg/m²) developed mild, non-infected AP with a 7-day hospitalization and no complications. The other patient (male, 60 years, BMI 28 kg/m²) developed INP confirmed by CT, with *E. coli* infection; he underwent EUS-guided necrosectomy, required ICU admission, and had a 20-day hospital stay without further complications.

### 3.2 Clinical characteristics and imaging

**Figure 2:**
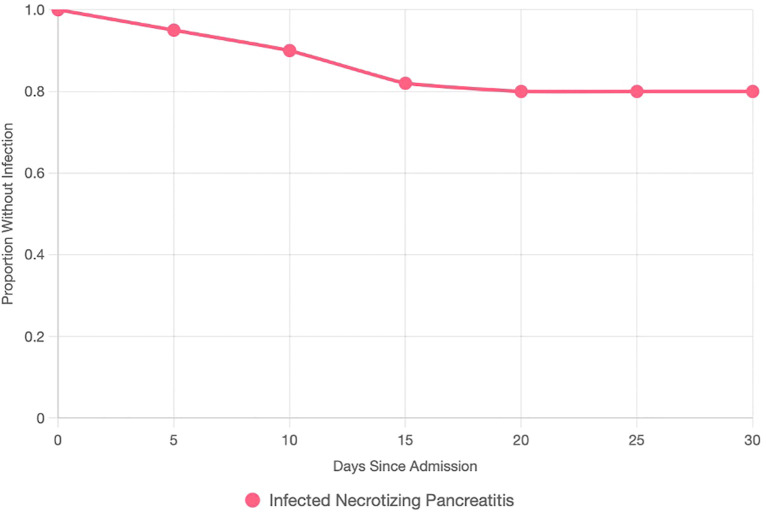
Kaplan–Meier survival curve plotting the proportion of AP patients without infected necrotizing pancreatitis (*y*-axis, 0–1) against days since admission (*x*-axis, 0–30). The curve declines, with a median infection onset at 12 days. Censored points (ticks) indicate patients without infection at study end or lost to follow-up.

Compared with non-infected cases, patients who developed infected AP were older (mean 60.1 ± 11.2 vs. 48.5 ± 14.9 years, *p* < 0.05), had higher BMI (≥25 kg/m² in 18/21 vs. 65/98, *p* = 0.03), and exhibited more severe disease (BISAP score ≥3 in 13/21 vs. 10/98, *p* < 0.01) (**Table 1**). Common presenting symptoms in INP included epigastric pain (21/21), nausea (18/21), and vomiting (17/21). Laboratory findings showed higher admission CRP levels in infected cases (172.4 ± 86.1 vs. 104.8 ± 96.2 mg/L, *p* = 0.06) and elevated lipase (9,300 ± 9,500 vs. 6,750 ± 8,650 U/L, *p* = 0.06), though differences did not reach conventional significance. Imaging confirmed necrosis in all infected cases, using CT (74/119) and US (70/119). Peripancreatic fluid collections (9/21 vs. 39/98, *p* = 0.79), fat stranding (9/21 vs. 44/98, *p* = 0.87), and pleural effusions (6/21 vs. 11/98, *p* = 0.04) were documented (**Table 1**). The timing of infection diagnosis varied, reflecting tertiary referrals, with a median onset of 12 days (**Figure 2**). Infected cases demonstrated longer hospital stays (20 vs. 7 days, *p* < 0.01), higher ICU admission rates (13/21 vs. 8/98, *p* < 0.01), and mortality (2/21 vs. 0/98). Cholecystectomy was performed in 38 of 59 gallstone-related cases (infected, 9/21, including 5/8 conservative, 3/4 drainage, and 1/5 EUS; non-infected, 29/50).

**Table 1:** Clinical characteristics and imaging findings of patients with acute pancreatitis (*n* = 119).

Characteristic	Infected necrosis (*n* = 21)	Non-infected (*n* = 98)	*P*-value
Age (years, mean ± SD)	60.1 ± 11.2	48.5 ± 14.9	<0.05
Sex (male, *n*)	11/21	43/98	0.48
BMI ≥25 kg/m² (*n*)	18/21	65/98	0.03
BISAP ≥3 (*n*)	13/21	10/98	<0.01
Etiology (*n*)			
Gallstone	9/21	50/98	0.49
GLP-1 agonist	1/21	1/98	0.22
Idiopathic	5/21	22/98	0.88
Autoimmune	3/21	7/98	0.28
Alcohol-related	3/21	4/98	0.08
Imaging findings (*n*)			
Pancreatic necrosis	21/21	26/98	<0.01
Peripancreatic fluid	9/21	39/98	0.79
Fat stranding	9/21	44/98	0.87
Pleural effusion	6/21	11/98	0.04
Hospital stay (days, median)	20	7	<0.01
ICU admission (*n*)	13/21	8/98	<0.01

### 3.3 Microbiology and antibiotics

Microbiological analysis of INP cases from FNA, drainage, or necrosectomy identified *E. coli* (9/21), *K. pneumoniae* (5/21), *Pseudomonas aeruginosa* (4/21), and *Staphylococcus aureus* (3/21), with MDR observed in 7/21 patients, defined per CLSI guidelines as resistance to at least one agent in three or more antimicrobial classes. Sterile collection techniques minimized contamination. Therapeutic antibiotics were administered to all infected patients (21/21), initially empirically with broad-spectrum coverage and subsequently tailored based on culture results. Agents used included cephalosporins (9/21), carbapenems (7/21), and metronidazole/piperacillin–tazobactam (5/21). Prophylactic antibiotics were given in 60/119 patients, mainly for severe non-infected AP (BISAP ≥ 3 or necrosis > 30%, *n* = 18) or concomitant conditions such as cholecystitis (*n* = 42). Regimens included cephalosporins (25/60), carbapenems (15/60), metronidazole/piperacillin–tazobactam (8/60), and other agents (12/60) (**Table 2**). While the KAUH protocol emphasizes culture-directed therapy, the majority of prophylactic courses were initiated by referring facilities or emergency departments before specialist consultation.

**Table 2:** Antibiotic use and interventions in acute pancreatitis patients (*n* = 119).

Parameter	Infected necrosis (*n* = 21)	Non-infected (*n* = 98)	Total (*n* = 119)
Antibiotics used (*n*)			
Therapeutic	21/21	0/98	21/119
Prophylactic	0/21	60/98	60/119
Antibiotics (therapeutic, *n*)			
Cephalosporins	9/21	–	9/21
Carbapenems	7/21	–	7/21
Metronidazole/piperacillin–tazobactam	5/21	–	5/21
Antibiotics (prophylactic, *n*)			
Cephalosporins	–	25/60	25/60
Carbapenems	–	15/60	15/60
Metronidazole/piperacillin–tazobactam	–	8/60	8/60
Others	–	12/60	12/60
Interventions (*n*)			
Conservative	8/21	0/98	8/119
Percutaneous drainage	4/21	0/98	4/119
EUS-guided	5/21	0/98	5/119
Laparoscopic necrosectomy	2/21	0/98	2/119
Open necrosectomy	2/21	0/98	2/119

### 3.4 Treatment and management

**Figure 3:**
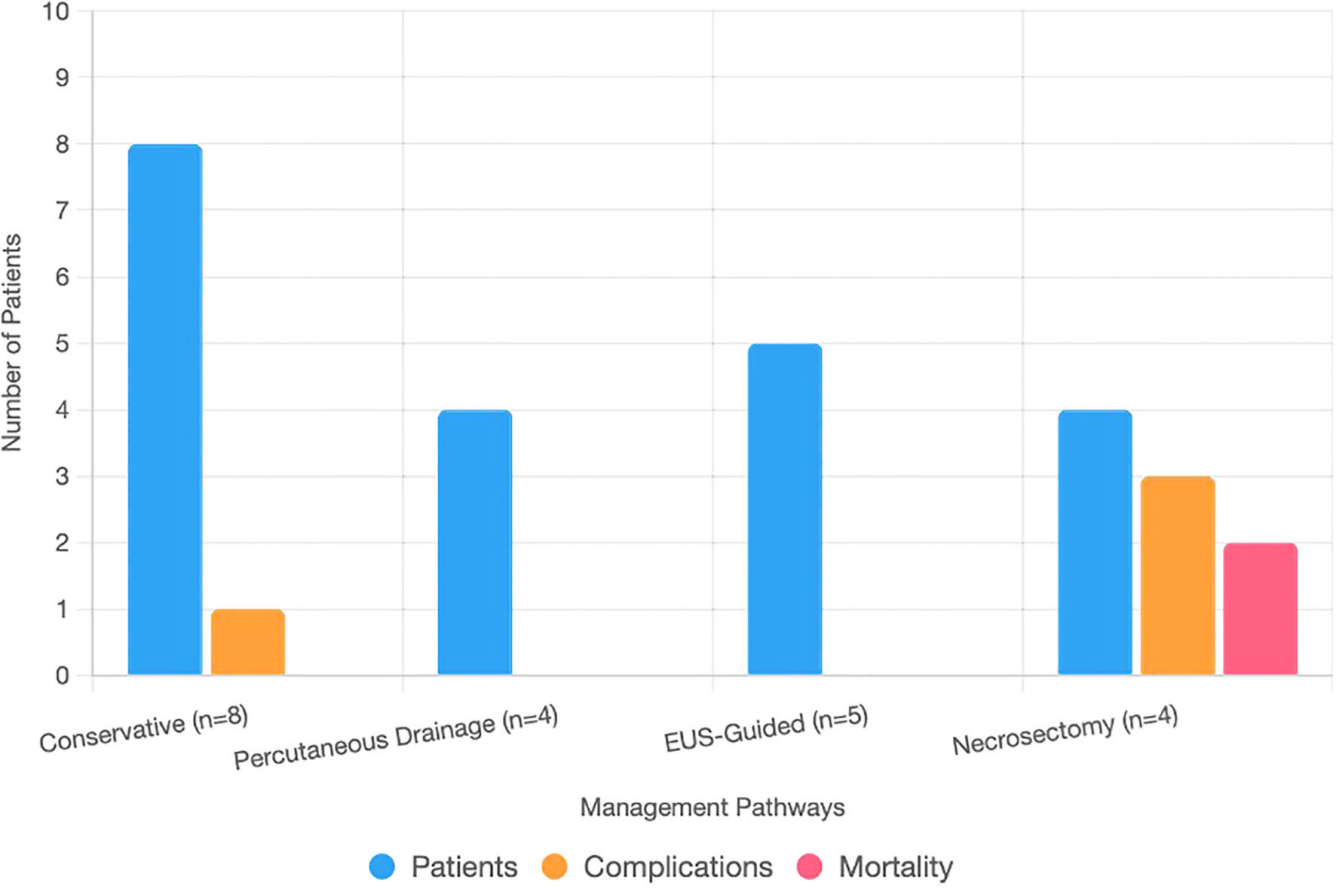
Bar chart showing patient counts, complications, and mortality across management pathways for infected necrotizing pancreatitis. The *x*-axis lists pathways: conservative (*n* = 8), percutaneous drainage (*n* = 4), EUS-guided (*n* = 5), and necrosectomy (*n* = 4). The *y*-axis shows counts (0–10). Stacked bars represent patients (blue), complications (orange: one fistula, one colonic perforation, two sepsis), and mortality (red: two deaths).

Among the 21 patients with INP, 8 were managed conservatively with antibiotics, fluid resuscitation, and early enteral nutrition when gastrointestinal function was intact. Four patients underwent percutaneous drainage for fluid-dominant necrosis occurring within 4 weeks of onset. Five patients underwent successful EUS-guided cystogastrostomy/necrosectomy for walled-off necrosis (>4 weeks). Four patients required necrosectomy after failed EUS due to inaccessible or solid necrosis, including two laparoscopic and two open procedures. Cholecystectomy was performed in 38 of 59 gallstone-related cases, including 9 of 21 infected patients (5 managed conservatively, 3 after percutaneous drainage, 1 following EUS), with concurrent cholecystectomy performed in all 4 patients undergoing necrosectomy. Enteral nutrition was administered in 16 of 21 infected cases (11 enteral alone, 5 combined with TPN). Eleven patients received TPN, either alone (*n* = 5) or in combination with enteral feeding, due to ileus or post-intervention requirements (**Table 2**; **Figure 3**).

### 3.5 Outcomes and complications

The median hospital stay for patients with INP was 20 days, with 13 of 21 requiring ICU admission. Among patients managed conservatively (*n* = 8), the median stay was 18 days, 5 required ICU care, and 1 developed a pancreatic fistula (95% CI 2.2%–47.1%). Percutaneous drainage (*n* = 4) was associated with a 16-day stay, two ICU admissions, and no recorded complications. EUS-guided cystogastrostomy or necrosectomy (*n* = 5) resulted in a 20-day median stay, three ICU admissions, and no major complications. Patients undergoing necrosectomy after failed EUS (*n* = 4) had a 28-day median stay, all required ICU care, and three experienced complications, including colonic perforation (1/2 laparoscopic, survived) and sepsis (1/2 laparoscopic, 1/2 open, both fatal). Overall mortality was 9.5% (2/21; 95% CI 2.4%–28.3%) (**Table 3**; **Figure 3**). Sensitivity analysis assuming worst-case outcomes for the 10 patients who were lost to follow-up (e.g., assuming additional mortality or complications) would increase mortality to 57.1%, with corresponding increases in complication rates; however, given that 8 of the 10 patients had at least 3 months of follow-up and only 2 had none, this likely influenced the estimates of long-term outcomes. Ten patients were lost to follow-up, likely due to residence outside Jeddah.

**Table 3:** Microbiology, antibiotics, and outcomes of infected necrotizing pancreatitis subgroups (*n* = 21).

Parameter	Conservative (*n* = 8)	Percutaneous drainage (*n* = 4)	EUS-guided (*n* = 5)	Necrosectomy (*n* = 4)
Microbiological profile (*n*)				
*E. coli*	4/8	2/4	2/5	2/4
*K. pneumoniae*	2/8	1/4	1/5	0/4
*P. aeruginosa*	1/8	0/4	2/5	2/4
*S. aureus*	1/8	1/4	0/5	0/4
Multidrug resistance	2/8	1/4	1/5	3/4
Antibiotics (therapeutic, *n*)				
Cephalosporins	4/8	3/4	2/5	0/4
Carbapenems	2/8	1/4	2/5	2/4
Metronidazole/piperacillin–tazobactam	2/8	0/4	1/5	2/4
Outcomes				
Hospital stay (days, median)	18	16	20	28
ICU admission (*n*)	5/8	2/4	3/5	4/4
Complications (*n*)	1 (fistula)	0	0	3 (1 perforation, 2 sepsis)
Mortality (*n*)	0/8	0/4	0/5	2/4

### 3.6 Predictors

Logistic regression identified age >60 years (OR 4.2; 95% CI 1.5–11.8; *p* < 0.05), BISAP score ≥3 (OR 7.8; 95% CI 2.6–23.1; *p* < 0.01), and hospital stay >10 days (OR 5.0; 95% CI 1.7–14.7; *p* < 0.01) as independent predictors of infection. Kaplan–Meier analysis demonstrated a median time to infection of 12 days (**Figure 2**).

## 4 Discussion

### 4.1 Epidemiology and predictors

In this cohort, 17.6% of AP cases developed INP, consistent with global estimates of 10%–20% (van Dijk et al., 2017). Gallstones (9/21) and GLP-1 receptor agonist exposure (1/21) reflect obesity-related etiologies, contrasting with alcohol-predominant cohorts in Western populations (Petrov and Yadav, 2019). GLP-1-associated pancreatitis, potentially related to ductal obstruction, may be underrecognized in the Middle East (Faillie et al., 2016). Independent predictors of infection included advanced age, BISAP score ≥3, and prolonged hospitalization, indicating that older patients with severe disease are at higher risk, in line with prior reports (Wu et al., 2008). Delayed presentation, frequently observed in tertiary referrals, may further contribute to longer hospital stays and increased complication rates.

### 4.2 Imaging

All INP cases demonstrated necrosis on CT, confirming the reliability of this modality for diagnosis (Baron et al., 2020). Findings such as peripancreatic fluid collections and pleural effusions were common but non-specific, suggesting that MRI may be considered for more complex cases or when conventional imaging is inconclusive (Zerem, 2014).

### 4.3 Microbiology and antibiotics

In this cohort, *E. coli* (9/21) and *K. pneumoniae* (5/21) predominated, with MDR observed in 7/21 cases, highlighting the challenges of MDR Enterobacteriaceae in the region (Memish et al., 2016). Higher rates of *P. aeruginosa* in patients with failed EUS interventions suggest that complex or solid necrosis may require broader-spectrum empiric coverage (Crocker et al., 2020). Therapeutic antibiotics were guided by culture results, whereas prophylactic use (60/119) exceeded typical Western rates (~30%, Mourad et al., 2021), primarily driven by severe disease (BISAP ≥ 3 or necrosis > 30%, 18/60), associated pathologies, or initiation at referring facilities before specialist consultation (42/60). KAUH’s protocol emphasizes culture-directed therapy and restricts prophylactic antibiotics, but tertiary referral patterns complicate adherence.

### 4.4 Management and outcomes

Conservative management was effective in stable INP cases, with a single pancreatic fistula reported (Mouli et al., 2015). Percutaneous drainage successfully managed abscesses (van Santvoort et al., 2011), while EUS-guided cystogastrostomy and necrosectomy were safe and complication-free (Arvanitakis et al., 2018). Patients requiring necrosectomy after failed EUS had high complication (3/4) and mortality rates (2/4), consistent with prior reports (Hollemans et al., 2016). Overall, the step-up management approach reduced morbidity (Boxhoorn et al., 2020), and cholecystectomy (38/59) likely mitigated recurrence risk (Moody et al., 2019). The overall mortality of 9.5% (95% CI 2.4%–28.3%) was below global averages (15%–30%) but underscores the substantial risk in severe or complicated cases (Leppäniemi et al., 2019). The high complication rate observed in necrosectomy cases may reflect referral bias toward severe cases at a tertiary care center. Furthermore, the non-standardized use of prophylactic antibiotics—primarily initiated by referring facilities—limits the ability to draw definitive conclusions about management effectiveness.

### 4.5 Non-antibiotic strategies

High prophylactic antibiotic use (60/119), particularly initiated prior to referral, suggests potential overuse. Early enteral nutrition, implemented in 16 of 21 infected cases, may mitigate infection risk and improve outcomes (Sun et al., 2013). The role of probiotics in modulating SIRS remains under investigation and warrants further study (Besselink et al., 2009).

### 4.6 Limitations

This study has several limitations. Its single-center, retrospective design and small cohort of infected patients (*n* = 21) restrict generalizability. Referral bias at KAUH, a tertiary care center, may have skewed the sample toward more severe cases, potentially overestimating complication and mortality rates. Missing follow-up data for 10 patients and variability in infection diagnosis timing due to external referrals reduce precision. Patient-centered outcomes, such as quality of life, were not assessed. Small subgroup sizes (*n* = 2–8) limited statistical power, necessitating combined analyses for some interventions. Additionally, generalizability to other Middle Eastern centers may be limited by differences in resources, referral patterns, and microbial prevalence.

## 5 Conclusion

In this cohort of 119 AP patients, 21 (17.6%) developed INP, managed through tailored interventions ranging from conservative care to necrosectomy. Complications, including pancreatic fistula, colonic perforation, and sepsis, occurred alongside a 9.5% mortality rate (95% CI 2.4%–28.3%), highlighting the substantial clinical risk. Gallstones remained the predominant etiology, while GLP-1 agonist use emerged as a potential contributor in obesity-prone populations. Advanced age, high BISAP scores, and prolonged hospitalization were significant predictors, and imaging guided timely intervention. High prophylactic antibiotic use (60/119) and MDR infections (7/21) underscore the need for antibiotic stewardship, whereas early enteral nutrition (16/21) may mitigate complications. This preliminary single-center study, limited by its small single-center design and referral bias, provides preliminary findings that require validation in larger studies focusing on regional microbial patterns, clinical outcomes, and management strategies. Multicenter, prospective studies with standardized protocols and extended follow-up are warranted to improve generalizability and optimize INP care in the Middle East.

## Data Availability

The raw data supporting the conclusions of this article will be made available by the authors, without undue reservation.
